# Role of fluorine-18-fluorodeoxyglucose positron emission tomography in selecting candidates for a minimally invasive approach for thymic epithelial tumour resection

**DOI:** 10.1093/icvts/ivad082

**Published:** 2023-05-19

**Authors:** Takaki Akamine, Kazuo Nakagawa, Kimiteru Ito, Hirokazu Watanabe, Masaya Yotsukura, Yukihiro Yoshida, Yasushi Yatabe, Masahiko Kusumoto, Shun-Ichi Watanabe

**Affiliations:** Department of Thoracic Surgery, National Cancer Center Hospital, Tokyo, Japan; Department of Thoracic Surgery, National Cancer Center Hospital, Tokyo, Japan; Department of Diagnostic Radiology, National Cancer Center Hospital, Tokyo, Japan; Department of Diagnostic Radiology, National Cancer Center Hospital, Tokyo, Japan; Department of Thoracic Surgery, National Cancer Center Hospital, Tokyo, Japan; Department of Thoracic Surgery, National Cancer Center Hospital, Tokyo, Japan; Department of Diagnostic Pathology, National Cancer Center Hospital, Tokyo, Japan; Department of Diagnostic Radiology, National Cancer Center Hospital, Tokyo, Japan; Department of Thoracic Surgery, National Cancer Center Hospital, Tokyo, Japan

**Keywords:** Thymic epithelial tumours, Fluorine-18-fluorodeoxyglucose positron emission tomography, Minimally invasive approach

## Abstract

**OBJECTIVES:**

We evaluated the potential of preoperative fluorine-18-fluorodeoxyglucose positron emission tomography to predict invasive thymic epithelial tumours in patients with computed tomography-defined clinical stage I thymic epithelial tumours ≤5 cm in size who are generally considered to be candidates for minimally invasive approaches.

**METHODS:**

From January 2012 to July 2022, we retrospectively analysed patients who exhibited tumour-node-metastasis (TNM) clinical stage I thymic epithelial tumours with lesion sizes ≤5 cm as determined by computed tomography. All patients underwent fluorine-18-fluorodeoxyglucose positron emission tomography preoperatively. We analysed the association of maximum standardized uptake values with both the World Health Organization histological classification and the TNM staging classification.

**RESULTS:**

A total of 107 patients with thymic epithelial tumours (thymomas, 91; thymic carcinomas, 14; carcinoids, 2) were evaluated. Nine patients (8.4%) were pathologically upstaged: TNM pathological stage II in 3 (2.8%), III in 4 (3.7%) and IV in 2 (1.9%). Among these 9 upstaged patients, 5 had thymic carcinoma with stage III/IV, 3 had type B2/B3 thymoma with stage II/III and 1 had type B1 thymoma with stage II. Maximum standardized uptake values were a predictive factor that distinguished pathological stage >I thymic epithelial tumours from pathological stage I [best cut-off value, 4.2; area under the curve = 0.820] and thymic carcinomas from other thymic tumours (best cut-off value, 4.5; area under the curve = 0.882).

**CONCLUSIONS:**

Thoracic surgeons should carefully determine the surgical approach for high fluorodeoxyglucose-uptake thymic epithelial tumours and keep in mind the issues associated with thymic carcinoma and potential combined resections of neighbouring structures.

**Figure:**
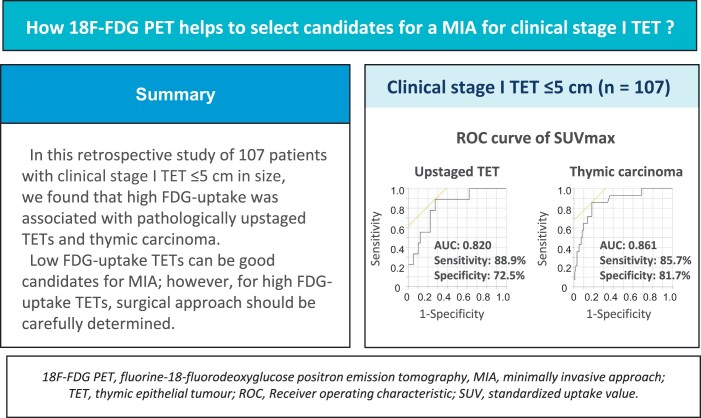

## INTRODUCTION

Thymic epithelial tumour (TET) is the most common neoplasm in the anterior mediastinum, consisting of thymoma and thymic carcinoma. Surgical resection is the gold standard treatment for resectable TETs [1]. The surgical procedure includes various approaches: minimally invasive approach (MIA), standard open (median sternotomy) approach and others; different extents of resection of the normal thymus: tumour resection without and with total thymectomy (thymomectomy and thymothymomectomy, respectively); and differences in the need for combined resection of neighbouring structures [2]. An MIA may include multi-portal video-assisted thoracic surgery (VATS), subxiphoid uniportal VATS, hybrid VATS and robotic-assisted thoracic surgery. This MIA is widely used in common practice for thoracic surgery. However, the National Comprehensive Cancer Network guidelines do not routinely recommend MIA for TETs due to the lack of long-term data [3].

In general, MIA is applied to non-invasive, or tumour-node-metastasis (TNM) clinical (c-) stage I, TETs ≤5 cm in size on computed tomography (CT) images (MIA-candidate TETs) [4, 5]. Even among such CT-determined non-invasive tumours, there are some invasive tumours or thymic carcinomas that might be unsuitable for an MIA. Moreover, although MIA has been reported to be both effective and feasible for early-stage thymomas [5], MIA for advanced-stage thymomas or thymic carcinoma is controversial because of its limited evidence. Therefore, the ability to predict thymic carcinoma or potentially invasive TETs that need to be resected with combined resection of neighbouring structures in MIA-candidate TETs should help thoracic surgeons determine the optimal surgical approach: MIA, open or other [6].

Over the past decade, many studies have shown that fluorine-18-fluorodeoxyglucose positron emission tomography (18F-FDG PET) helps determine the World Health Organization (WHO) histological classification, especially for thymoma versus thymic carcinoma, and predicts invasive TETs [7–9]. However, given that TETs are rare, only a few studies with insufficient sample sizes have been conducted on preoperative CT-determined MIA-candidate TETs. Therefore, we investigated the diagnostic role of 18F-FDG PET for predicting invasive tumours and histologic subtypes in MIA-candidate TETs.

## MATERIALS AND METHODS

### Ethical statement

This study was approved by our institutional review board (approval number: 2018-45, approval date: 25 May 2018), which waived the requirement for informed consent based on the study’s retrospective observational design.

### Patient selection

Between January 2012 and July 2022, 222 consecutive patients underwent thymectomy at the Department of Thoracic Surgery of National Cancer Center Hospital, Tokyo, Japan. The inclusion criteria were c-stage I TETs ≤5 cm in size on CT images. Exclusion criteria included recurred patients; patients who received preoperative treatment or palliative treatment; and patients who did not undergo preoperative 18F-FDG PET. We reviewed the patients’ medical records and their radiological, operative and pathological findings. Tumour size was evaluated based on the maximum diameter measured by CT.

### Preoperative examination, tumour staging and histological subtype

All patients underwent chest radiography and contrast-enhanced CT. Either positron emission tomography (PET)/CT or PET/magnetic resonance imaging (MRI) was performed within approximately 3 months before surgery. In routine clinical practice, preoperative pathological diagnosis is usually omitted for an anterior mediastinal lesion that is suspected to be resectable thymoma in preoperative CT examination. Clinical, surgical (s-) and pathological (p-) staging was performed according to the 8th edition of the Union for International Cancer Control tumour-node-metastasis classification of malignant tumours [10, 11]. The criterion for clinical lymph node (LN) metastasis was short-axis diameter larger than 1 cm, or a node with fluorodeoxyglucose (FDG) uptake by 18F-FDG PET. The staging was defined by reviewing the medical records and pathological and radiological reports. In our institution, the criteria for determining invasion (clinical T2, T3 and T4) on CT were irregularity of the tumour contour and a lack of fat planes between the tumour and surrounding structures. The histological type was diagnosed based on resected specimens according to the WHO classification [12]. Thymoma was classified as low-risk thymoma (types A, AB and B1) and high-risk thymoma (types B2 and B3) based on supported evidence [13]. Micronodular thymoma and metaplastic thymoma were classified as low-risk thymoma.

### Preoperative fluorine-18-fluorodeoxyglucose positron emission tomography

Patients fasted for at least 4 h before an intravenous injection of 3–4 MBq/kg of 18F-FDG. Patients rested for ∼1 h before image acquisition with Discovery 600 PET/CT scanners or a SIGNA PET/MRI scanner (GE Medical Systems, Milwaukee, WI, USA). Data from three-dimensional emission scanning were reconstructed by iterative ordered subset expectation maximization. The tumour 18F-FDG PET data were evaluated semiquantitatively based on maximum standardized uptake values (SUVmax). A region of interest was placed over the tumour after correcting for radioactive decay to measure SUVmax. The MRI sequence for PET/MRI was acquired as we previously reported [9].

### Treatment strategies, surgical procedures and postoperative follow-up

For a lesion 5 cm or less in size without any findings of invasion to surrounding structures, thymomectomy through MIA is usually performed [14]. We performed MIA by VATS with 3 ports (complete VATS) or VATS with mini-thoracotomy (hybrid VATS) (see more detail in the Supplementary Material, Text) [14]. On the other hand, median sternotomy was performed if the tumour was close to the innominate vein. In addition, thymothymomectomy was performed through median sternotomy in patients with myasthenia gravis. Thymomectomy (or thymothymomectomy) with combined resection was performed when direct invasion by tumour was suspected. Extended LN dissection was not performed, but LN sampling was performed for visible LN adjacent to the primary tumour. Postoperatively, patients were followed up every 6 months for the first 2 years after the operation and once yearly thereafter. A physical examination and chest radiography were performed every 6 months, and a chest CT examination was performed every year.

### Statistical analysis

The outcomes of interest are the differences in SUVmax between pathologically upstaged TETs and p-stage I TETs and between thymic carcinoma and thymoma. Categorical variables are expressed as numbers and percentages, and continuous data are described as medians and ranges. Groups were compared using the Mann–Whitney *U* test for continuous variables. The cut-off values of SUVmax for differentiating thymic carcinoma from other TETs, and pathologically upstaged patients from p-stage I patients, were determined by receiver operating characteristic (ROC) curve analysis. Statistical analyses were performed with JMP software, version 14 (SAS Institute, Cary, NC, USA). *P*-values <0.05 were considered statistically significant.

## RESULTS

### Patient characteristics of minimally invasive approach-candidate thymic epithelial tumours

**Figure 1:**
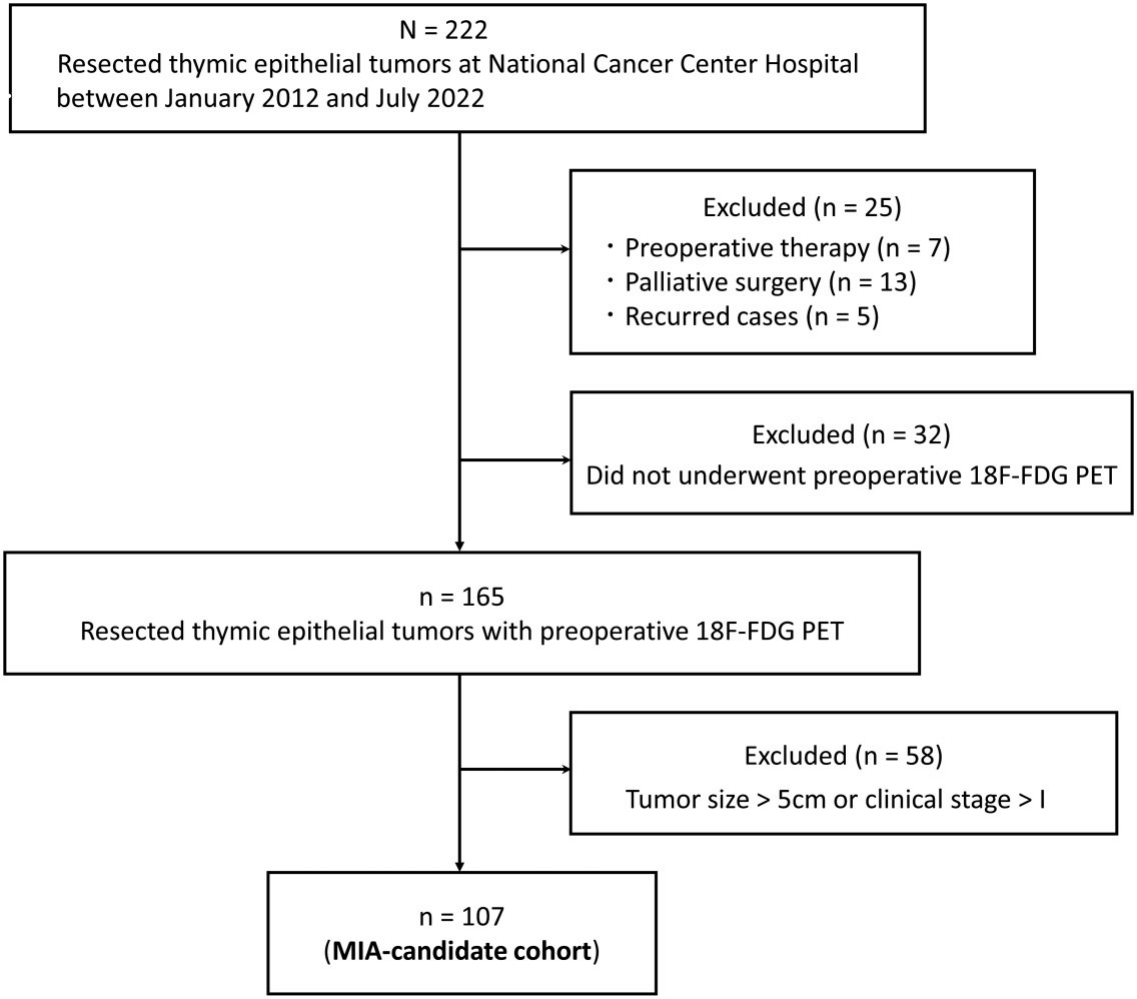
Flow chart illustrating the study design. MIA: minimally invasive approach

A total of 107 patients with TNM c-stage I TETs ≤5 cm in size on CT images were included in the analysis (Fig. 1). The clinical characteristics of patients with MIA-candidate TETs are summarized in Table 1. According to the WHO classification and the risk stratification of thymoma, 91 patients were diagnosed with thymoma (69 with high-risk, 22 with low-risk thymoma), 14 with thymic carcinoma and 2 with carcinoid. The median tumour size was 3.3 cm (range: 1.1–5.0 cm). Complete VATS was performed on 53 patients, hybrid VATS on 33 and median sternotomy on 21. Thymomectomy (or thymothymomectomy) with combined resection, which corresponds to s-stage >I, was performed on 18 (16.8%) patients. Regarding CT findings, 96.2% of patients had either tumours with a smooth contour (*n* = 95) or tumours with a lobular contour (*n* = 8). Four patients had a tumour with an irregular contour (high-risk thymoma, *n* = 1; thymic carcinoma, *n* = 3). Of 9 pathologically upstaged patients, 3 (33%) had a tumour with an irregular contour and 6 (67%) had a tumour with a smooth contour.

**Table 1:** Characteristics of patients with minimally invasive approach-candidate thymic epithelial tumours

Factors	MIA-candidate patients with TNM clinical stage I TETs			*P*-Value
	Overall	Upstaged patients	Pathological stage I patients	
	*n* = 107	*n* = 9	*n* = 98	
Age (years), median (range)	62 (26–84)	62 (26–84)	57 (39–75)	0.42
Sex, *n* (%)				0.72
Male	49 (45.8)	5 (55.6)	44 (44.9)	
Female	58 (54.2)	4 (44.4)	54 (55.1)	
WHO classification				<0.001
Low-risk thymoma^a^ (type A, AB, B1)	69 (64.5)	1 (11.1)	68 (69.4)	
High-risk thymoma (type B2, B3)	22 (20.6)	3 (33.3)	19 (19.4)	
Thymic cancer	14 (13.1)	5 (55.6)	9 (9.2)	
Carcinoid	2 (1.9)	0 (0.0)	2 (2.0)	
Tumour size (cm), median (range)	3.3 (1.1–5.0)	3.6 (2.5–4.3)	3.3 (1.1–5.0)	0.42
SUVmax, median (range)	3.7 (1.0–15.7)	5.6 (3.1–15.7)	3.6 (1.0–9.8)	0.002
Surgical approach, *n* (%)				0.018
Complete VATS	53 (49.5)	1 (11.1)	52 (53.1)	
Hybrid VATS	33 (30.9)	4 (44.4)	29 (29.6)	
Median sternotomy	21 (19.6)	4 (44.4)	17 (17.3)	
Surgical procedure, *n* (%)				
Thymomectomy (or thymothymomectomy)	89 (83.2)	0 (0.0)	89 (90.8)	
Thymomectomy (or thymothymomectomy) with combined resection^b^	18 (16.8)	9 (100.0)	9 (9.2)	
Pericardium resection	13 (12.1)	7 (77.8)	6 (6.1)	
Lung resection	6 (5.6)	4 (44.4)	2 (2.0)	
Phrenic nerve resection	4 (3.7)	3 (33.3)	1 (1.0)	
Innominate vein resection	2 (1.9)	2 (22.2)	0 (0.0)	

a Micronodular thymoma and metaplastic thymoma are included. Data are shown as *n* (%) or median (range).

b Data overlapping (thymomectomy or thymectomy was extended to multiple neighbouring organs in 5 cases).

MIA: minimally invasive approach; SUVmax: maximum standardized uptake value; TET: thymic epithelial tumour; VATS: video-assisted thoracic surgery; WHO: World Health Organization.

### Clinical, surgical and pathological characteristics of pathologically upstaged patients

**Figure 2:**
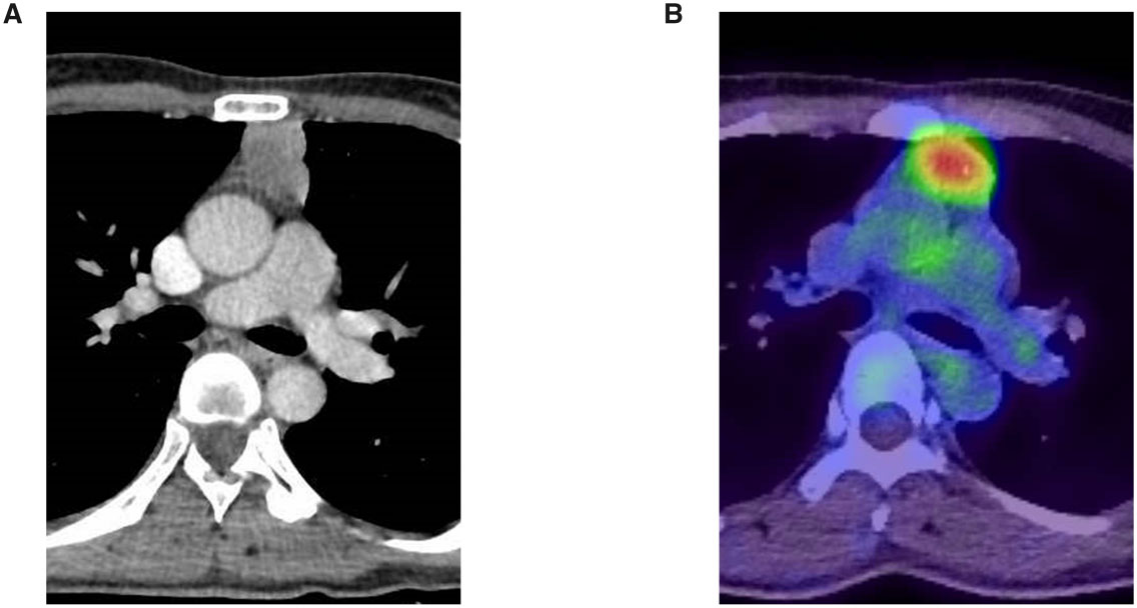
Representative images of clinical stage I type B3 thymoma that required combined resection of the innominate vein: (**A**) axial enhanced CT demonstrated that the maximum tumour diameter was 3.6 cm. (**B**) 18F-FDG PET/CT image shows high FDG uptake within the mass with SUVmax of 5.6. CT: computed tomography; 18F-FDG PET: fluorine-18-fluorodeoxyglucose positron emission tomography; SUVmax: maximum standardized uptake value.

Pathological upstaging occurred in 8.4% (*n* = 9) of patients with c-stage I TET ≤5 cm in size. There was no significant difference in age, sex or tumour size between the upstaged and p-stage I patients (*P* = 0.416, 0.723 and 0.422, respectively, Table 1). Of the 9 upstaged patients, 5 had thymic carcinoma, 3 had high-risk thymoma (type B2, *n* = 1; B3, *n* = 2) and 1 had low-risk thymoma (type B1, *n* = 1). The mean SUVmax was significantly higher in upstaged patients than in p-stage I (*P* = 0.002, Table 1). Figure 2 shows representative CT and 18F-FDG PET/CT images of a patient with type B3 thymoma invading the innominate vein, which was clinically diagnosed as non-invasive tumour. Eighteen patients were considered to be surgically upstaged, but pathological invasion was observed in 8 patients; 3 patients (2.8%) had tumours involved with pericardium (pT2); and 5 (4.7%) had tumours involved with lung (*n* = 3) or innominate vein (*n* = 2, pT3, Table 2). Two patients with high FDG-uptake thymic carcinoma (SUVmax = 6.1, 15.7, respectively) had LN metastasis in anterior peritumoural LN (pN1). There was no conversion from MIA to the open approach.

**Table 2:** Pathological and surgical stage according to the 8th edition of the Union for International Cancer Control tumour-node-metastasis classification

TNM classification	Clinical stage I thymic epithelial tumours	
	Surgical stage	Pathological stage
Tumour stage, *n* (%)		
T1	89 (83.2)	99 (92.5)
T2	8 (7.5)	3 (2.8)
T3	10 (9.3)	5 (4.7)
Nodal stage, *n* (%)		
N0	107 (100)	105 (98.1)
N1	0 (0)	2 (1.9)
Distant metastasis, *n* (%)		
M0	107 (100.0)	107 (100.0)
TNM stage, *n* (%)		
I	89 (83.2)	98 (91.6)
II	8 (7.5)	3 (2.8)
III	9 (8.4)	4 (3.7)
IVA	1 (0.9)	2 (1.9)

Data are shown as *n* (%).

MIA: minimally invasive approach; TNM: tumour-node-metastasis.

### Fluorine-18-fluorodeoxyglucose positron emission tomography *evaluation in* minimally invasive approach*-candidate thymic epithelial tumours*

**Figure 3:**
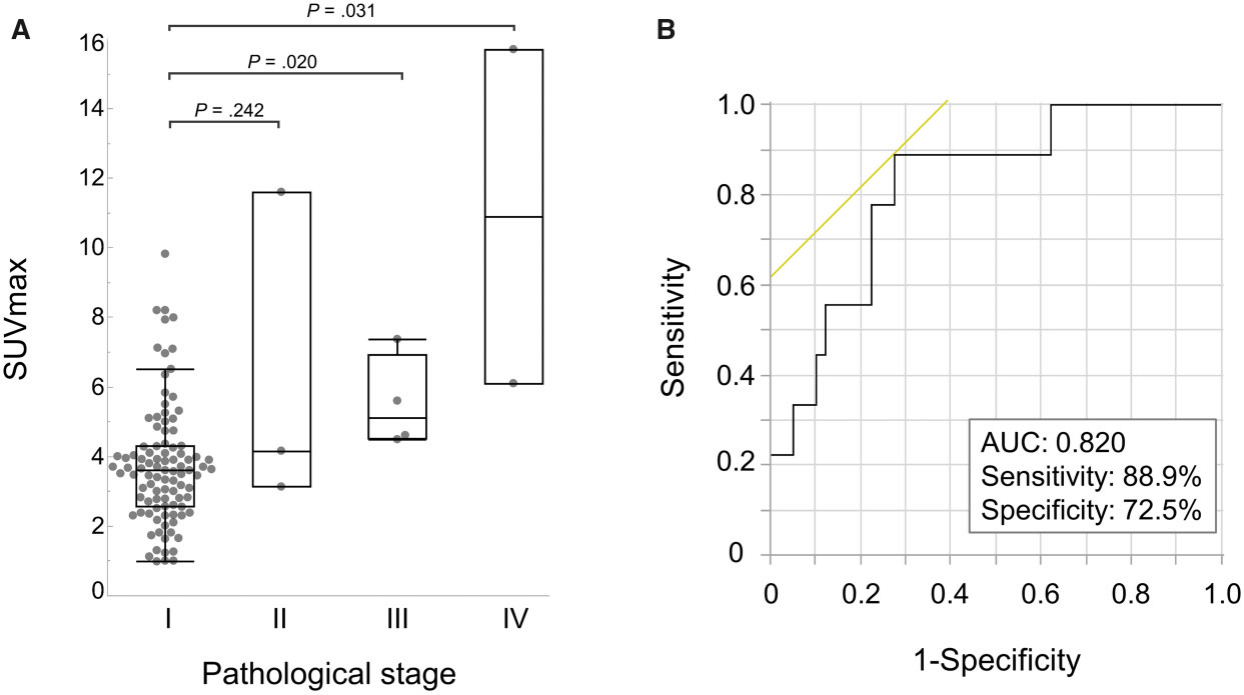
Box plots of SUVmax according to pathological TNM stage (**A**) and area under the ROC curve for detecting advanced stage (**B**). 18F-FDG PET: fluorine-18-fluorodeoxyglucose positron emission tomography; MIA: minimally invasive approach; ROC: receiver operating characteristic; SUVmax: maximum standardized uptake value; TNM: tumour-node-metastasis.

**Figure 4:**
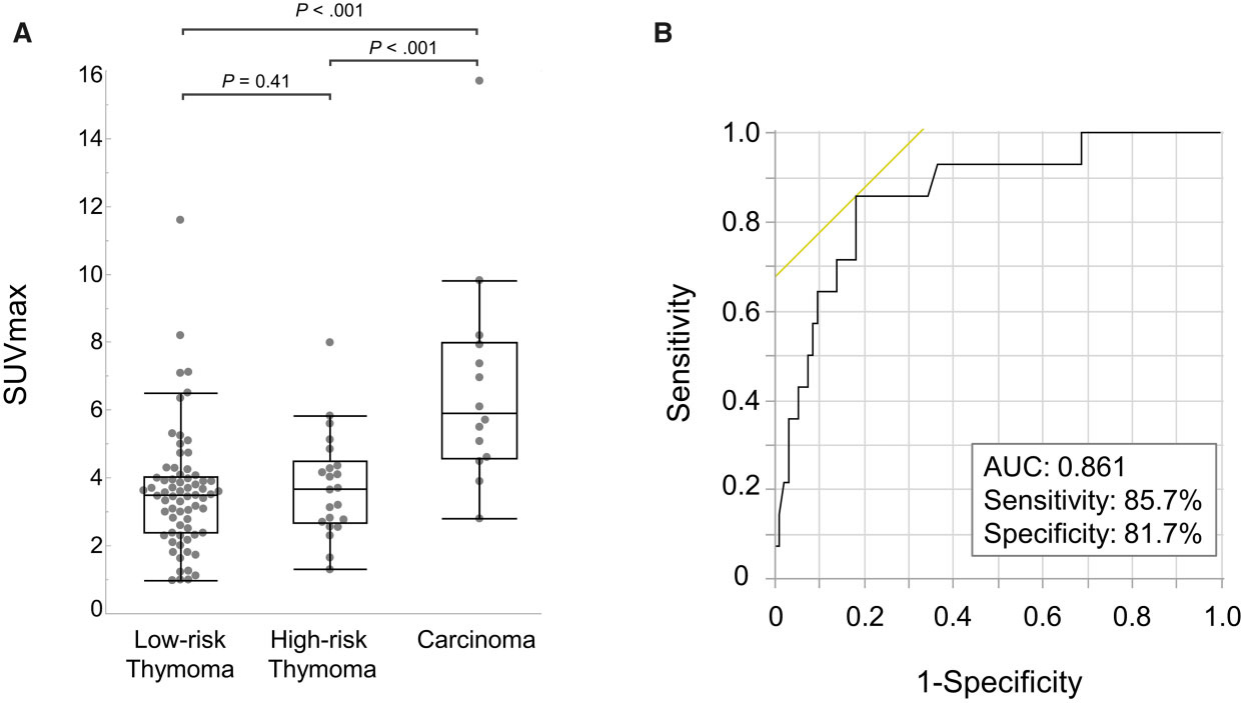
Box plots of SUVmax according to World Health Organization histological classification in the MIA-candidate cohort (**A**). Area under the ROC curve for detecting thymic carcinoma in the MIA-candidate cohort (**B**). 18F-FDG PET: fluorine-18-fluorodeoxyglucose positron emission tomography; MIA: minimally invasive approach; ROC: receiver operating characteristic; SUVmax: maximum standardized uptake value.

The distribution of SUVmax in patients stratified according to TNM staging is demonstrated in Fig. 3A. 18F-FDG uptake in the tumour tended to gradually increase from TNM p-stage I to IV. The ROC analysis demonstrated that the area under the curve was 0.820 (95% confidence interval, 0.646–0.919), and the best cut-off value of SUVmax to differentiate the diagnosis of upstaged and p-stage I patients was 4.2 (sensitivity, 88.9%; specificity, 72.5%; Fig. 3B). The mean SUVmax of thymic carcinoma was significantly higher than that of thymoma (*P* < 0.001, Fig. 4A). There was no significant difference between the mean SUVmax of high-risk and low-risk thymoma (*P* = 0.41, Fig. 4A). According to the ROC analysis, SUVmax was a good predictor for the differential diagnosis of thymic carcinoma and others, and the best cut-off value of SUVmax was 4.5 (area under the curve, 0.861 [95% confidence interval, 0.721–0.937]; sensitivity, 85.7%; specificity, 81.7%; Fig. 4B).

### Surgical and pathological stages stratified by the simplified World Health Organization classification

**Figure 5:**
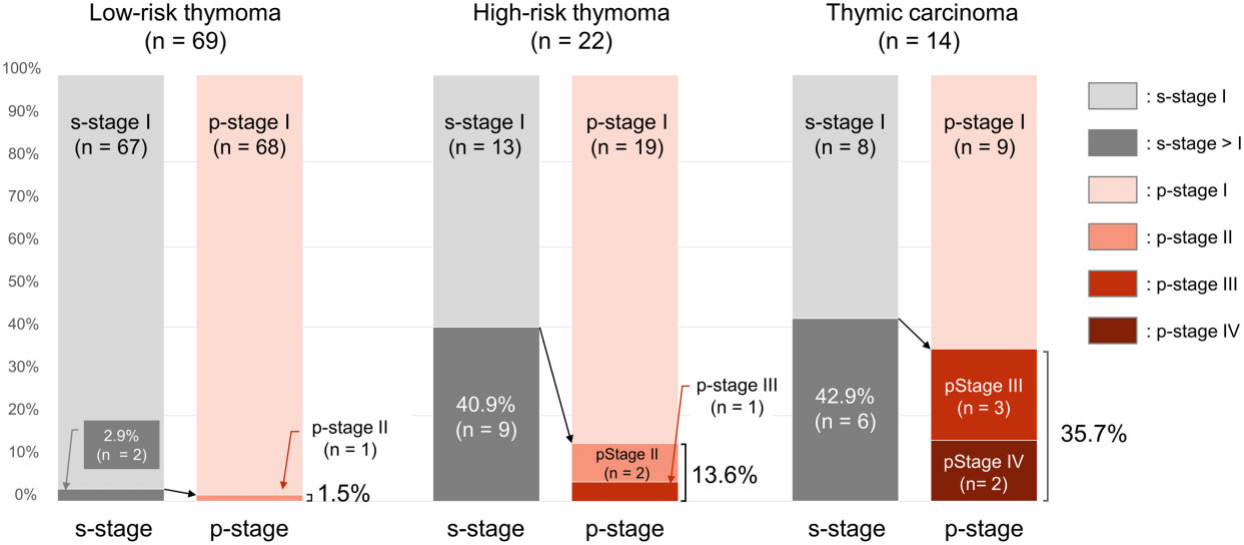
The proportion of surgically and pathologically upstaged patients in clinical stage I thymic epithelial tumours stratified by the simplified World Health Organization classification.

Patients who were suspected to be upstaged at surgery were more frequently seen in thymic carcinoma and high-risk thymoma than in low-risk thymoma (42.9% and 40.9% vs 2.9%, respectively, Fig. 5). In contrast, the proportion of pathologically upstaged patients was higher for thymic carcinoma, high-risk thymoma and low-risk thymoma (35.7%, 13.6% and 1.5%, respectively). A difference between surgical and pathological stages was observed in high-risk thymoma; one-third of patients who were suspected to be upstaged at surgery were pathologically upstaged.

### Surgical procedure and postoperative outcome of patients with clinical stage I thymic carcinoma

MIA was indicated in 11 patients, while the open approach was indicated in 3 patients. LN dissection was omitted for 9 patients with MIA, while LN sampling was performed on 5 patients (Table 3). Postoperative treatment was performed on 1 patient by radiation therapy with pathological N1. The median follow-up duration for patients with clinical stage I thymic carcinoma was 2.3 years (range: 0.5–7.3 years). LN metastasis did not occur in any of the patients during follow-up, while 2 recurred, 1 by loco-regional recurrence and bone metastasis and the other by pulmonary metastasis and dissemination. The cumulative incidence of recurrence is shown in Supplementary Material, Fig. S1.

**Table 3:** Surgical procedure and postoperative outcome of patients with clinical stage I thymic carcinoma

Factor	Patients with thymic carcinoma, *n* = 14
Surgical procedure	
Complete VATS	4 (28.6)
Hybrid VATS	7 (50.0)
Median sternotomy	3 (21.4)
Lymph node dissection	
Sampling	5 (35.7)
No	9 (64.3)
Median follow-up	2.3 (0.5–7.3)
Postoperative radiation therapy	1 (7.1)
Recurrence	2^a^ (14.3)
Lymph node metastasis	0 (0.0)

Data are shown as *n* (%) or median (range).

a One with both loco-regional recurrence and distant bone metastasis, and another with < pulmonary metastasis and dissemination.

VATS: video-assisted thoracic surgery.

## DISCUSSION

The use of MIA for TETs has increased with advances in technology and the improvement of minimal device and surgery techniques. Moreover, increased use of screening for lung cancer by low-dose CT has increased the incidental detection of small thymoma [15]. A recent analysis of early-stage TETs demonstrated the superiority of MIA over the open approach in postoperative outcomes and the non-inferiority of oncological outcomes between MIA and the open approach [16]. Therefore, the use of MIA for TETs will be further expanded. In the era of MIA, we have demonstrated that low FDG-uptake TETs are suitable candidates for MIA. In contrast, the thoracic surgeon should be careful in the selection of MIA for high FDG-uptake TETs because of the risk of tumour invasion into the surrounding tissue. We also found that no LN recurrence was observed after thymomectomy without extensive LN dissection in c-stage I thymic carcinoma ≤5 cm in size PET-negative LN, suggesting the possibility that LN dissection can be omitted in limited cases.

18F-FDG PET was a useful tool for predicting pathologically upstaged TETs in c-stage I TETs 5 cm or less in size on CT images. Thymic epithelial tumours less than 5 cm in size are usually considered to be candidates for MIA [5, 6]. The main reason to support this indication is that large-tumour handling by VATS is technically challenging to avoid capsule injury. Indeed, pleural recurrence caused by intraoperative capsule injury was observed after VATS thymectomy for TETs >5 cm in size, which led to a higher frequency of recurrence than for those ≤5 cm in size [17]. In addition to size criteria for MIA, an increasing body of evidence supports MIA for TNM clinical stage I (Masaoka stages I–II) since MIA achieved prognostic outcomes equivalent to those with the open approach [16]. In clinical practice, even if a patient is determined to be a good candidate for MIA according to size criteria and radiological findings, some patients may show unexpected progressed TETs at surgery. In the present study, 9 patients (8.4%) were pathologically upstaged in clinical stage I TETs. It is well known that there is a limit to the ability to predict the invasive potential TETs based on preoperative CT findings, and this can be even more difficult for tumours <5 cm [18]. Therefore, the ability to predict such occult invasive tumours by 18F-FDG PET preoperatively should help the surgeon to determine the surgical approach and plan the surgery to avoid technical misadventures, promote the safety of surgery and improve surgical outcomes. In addition, some information on the possibility of conversion from MIA to an open approach or combined resection of neighbouring structures can be provided to patients before surgery.

Fourteen patients (13%) had thymic carcinoma in MIA-candidate TETs. Among these 14 patients with thymic carcinoma, 35.7% (*n* = 5) were pathologically upstaged. Although MIA for thymectomy has been widely accepted, there is little evidence to support MIA for thymic cancer because MIA is indicated in a minority of patients with thymic carcinoma [19]. Moreover, the evidence supporting routine LN dissection is also insufficient and controversial, as National Comprehensive Cancer Network guidelines do not mention the indications for LN dissection [3]. In patients with MIA-candidate TETs, the diagnosis of thymic carcinoma by intraoperative frozen section is necessary to perform extended LN dissection; however, it is challenging to diagnose thymic carcinoma by intraoperative frozen section [20]. We hypothesized that LN dissection is unnecessary in patients with MIA-candidate TETs. In our MIA-candidate cohort study, MIA without LN dissection was performed in 9 patients. Of these 9, only 1 patient recurred, both loco-regionally and with distant bone metastasis. Moreover, LN metastasis was not observed in 2 patients with p-N1 (median follow-up; 5 years). Preoperatively undetectable LN metastasis was adjacent to the primary tumour; therefore, metastatic LN was dissected without performing extended LN dissection. We speculated that significantly increased FDG uptake by the primary tumour masked the detection of LN metastasis. It is too early to suggest an appropriate surgery for MIA without extended LN dissection for c-stage I thymic carcinoma ≤5 cm in size with PET-negative LN due to the small number of patients; therefore, further prospective or multicentre studies are warranted to investigate the prognosis of thymic carcinoma diagnosed after thymomectomy by MIA without LN dissection.

### Limitations

There are several potential limitations in this study. First, this was a retrospective study from a single institution experience and was susceptible to a selection bias. Although our study included a relatively large number of patients with TNM c-stage I TETs compared to previous studies, there were still not enough patients who were pathologically upstaged for analysis. Additional large prospective and multicentre studies are warranted to validate our findings and determine the appropriate cut-off value of SUVmax. Second, the postoperative follow-up of patients with thymic carcinoma is not long enough to support omitting extended LN dissection.

## CONCLUSION

Preoperative 18F-FDG PET was helpful in predicting pathologically upstaged TETs and thymic carcinoma in MIA-candidate TETs. Low FDG-uptake tumours were suitable candidates for MIA; however, for high FDG-uptake tumours, thoracic surgeons should be aware of the possible necessity of combined resection and carefully determine the surgical approach accordingly.

## Supplementary Material

ivad082_Supplementary_Data

## Data Availability

The data will be shared on reasonable request to the corresponding author.

## Notes

Presented at an e-poster session of the Fifty-ninth Annual Meeting of The Society of Thoracic Surgeons, San Diego, CA, USA, 21–24 January 2023.

## ABBREVIATIONS

CT: Computed tomography
FDG: Fluorodeoxyglucose
18F-FDG PET: Fluorine-18-fluorodeoxyglucose positron emission tomography
LN: Lymph node
MIA: Minimally invasive approach
MRI: Magnetic resonance imaging
PET: Positron emission tomography
ROC: Receiver operating characteristic
SUVmax: Maximum standardized uptake value
TET: Thymic epithelial tumour
TNM: Tumour-node-metastasis
VATS: Video-assisted thoracic surgery
WHO: World Health Organization
