# Supplementary material for: Role of fluorine-18-fluorodeoxyglucose positron emission tomography in selecting candidates for a minimally invasive approach for thymic epithelial tumour resection
Source: Interdiscip Cardiovasc Thorac Surg. 2023 May 19;36(5):ivad082. doi: 10.1093/icvts/ivad082 (PMC10232332; doi:10.1093/icvts/ivad082)
Supplement: ivad082_Supplementary_Data [file ivad082_supplementary_data.zip › Supplementary Text.docx]

**Supplementary Text**

*Surgical approach:* *video-assisted thoracic surgery*

Briefly, we perform complete video-assisted thoracic surgery (VATS) by 3 ports without CO_2_ insufflation or sternal lifting. We place the camera port in the 5th intercostal space (ICS) along the middle axillary line. The other two ports are placed in the 3rd or 4th ICS at the anterior or middle axillary line where appropriate. An access window is made from a 2.5cm skin incision, and a wound protector is placed on it. The resected thymus with tumour is removed through the access window with a plastic retrieval bag. On the other hand, VATS with mini-thoracotomy (hybrid VATS) is performed by muscle-sparing anterior thoracotomy (3rd or 4th ICS with a 3-4cm incision) and a camera port (5th ICS).

**Supplementary Figure 1**

Cumulative incidence of recurrence in patients with clinical stage I thymic carcinoma
