# Supplementary figures and images for: Role of fluorine-18-fluorodeoxyglucose positron emission tomography in selecting candidates for a minimally invasive approach for thymic epithelial tumour resection
Source: Interdiscip Cardiovasc Thorac Surg. 2023 May 19;36(5):ivad082. doi: 10.1093/icvts/ivad082 (PMC10232332; doi:10.1093/icvts/ivad082)

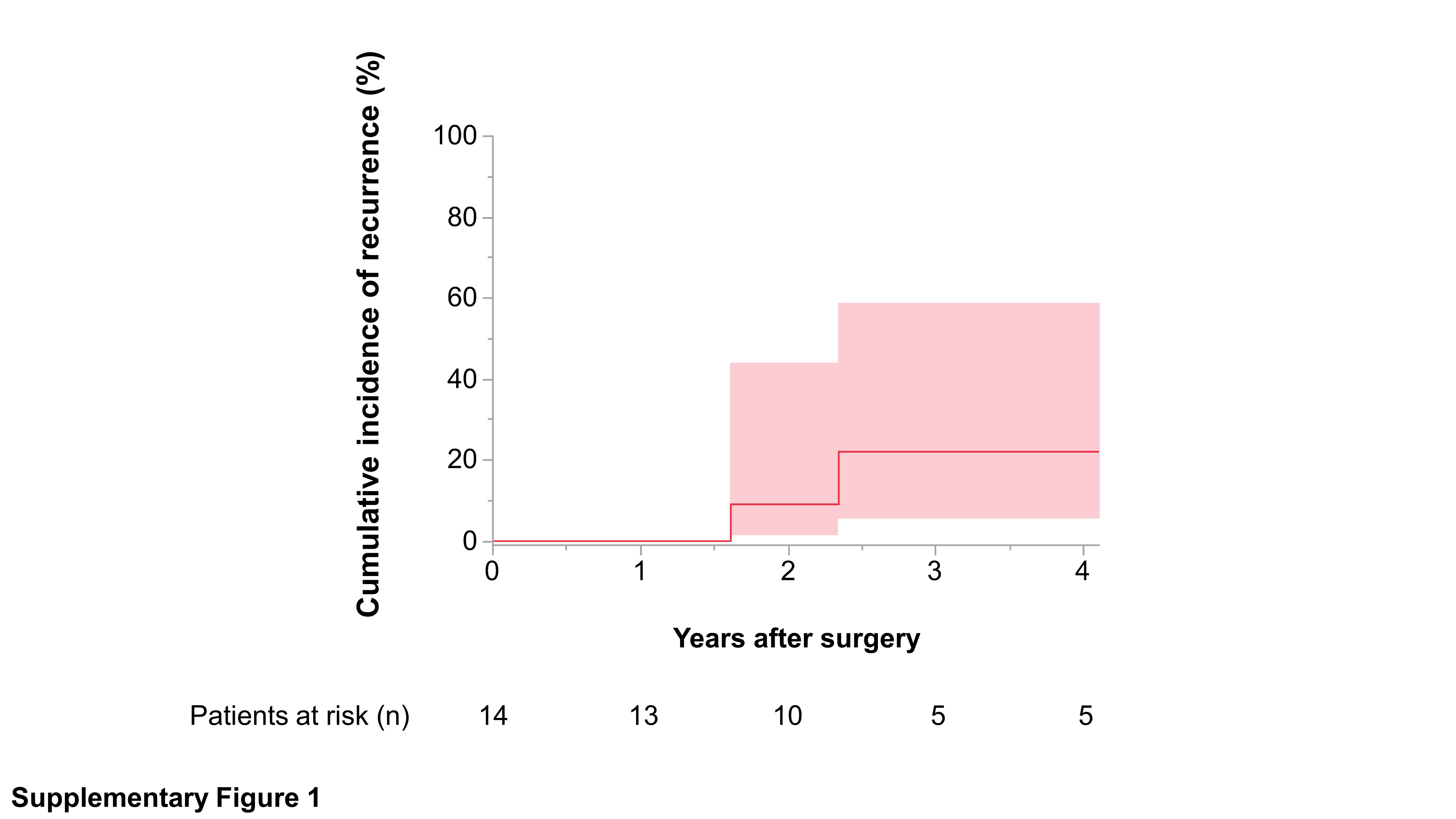

Supplement: ivad082_Supplementary_Data [file ivad082_supplementary_data.zip › Supplementary Figure 1.TIF]
